# Crosslinking Surgical Oncology and the Assessments of Hernia Sac Tissues With Malignant Transformations

**DOI:** 10.7759/cureus.84317

**Published:** 2025-05-18

**Authors:** Lareb Asad, Mostafa Ahmed Abdellah Ahmed, Madeeha Minhas, Durga Devi, Seemi Tanvir, Waqar Azeem, Muhammad Khaliq, Muhmmad Hussain Shah

**Affiliations:** 1 Department of Pathology, People's University of Medical and Health Sciences for Women, Nawabshah, PAK; 2 Department of General Surgery, Frimley Health NHS Foundation Trust, Frimley, GBR; 3 Department of Pathology, College of Science and Health Professions, King Saud Bin Abdulaziz University for Health Sciences, Jeddah, SAU; 4 Department of Pathology, Liaquat University of Medical and Health Sciences, Jamshoro, PAK; 5 Department of Pathology, Margalla Institute of Health Sciences, Rawalpindi, PAK; 6 Department of Pathology, Isra University, Hyderabad, PAK; 7 Department of Pathology, University of Health Sciences, Lahore, PAK; 8 Department of Pathology, Dow University of Health Sciences, Dow International Medical College, Karachi, PAK; 9 Department of Molecular Pathology, University of the Punjab, Lahore, PAK

**Keywords:** hernia sac, malignant transformation, mesothelial changes, pre-neoplastic lesions, surgical oncology

## Abstract

Background: Hernia repairs are performed frequently, yet studies investigating the presence of malignancies within hernia sac tissues during these operations remain limited. The research investigated histopathological changes in hernia sac tissues, along with early signs of malignancy, while integrating surgical oncology practices with diagnostic pathological examinations.

Methods: This prospective observational study included 100 adult patients who underwent abdominal surgery between January and September 2022. Eighty candidates undergoing elective hernia repair surgery participated in this study, along with 20 patients with age and sex similarities undergoing unrelated abdominal surgeries. Sample size was calculated using OpenEpi software version 3.0.0 (Dean, Sullivan, Soe: Atlanta, GA). General evaluation of the hernia sac tissue involved the examination of chronic inflammation, mesothelial hyperplasia, atypical cellular changes, fibrosis, and vascular proliferation. Statistical analysis was done using SPSS version 26 (Armonk, NY: IBM Corp.) with a p-value of <0.05 considered statistically significant.

Results: Out of the total participants in the hernia group, 34 (42.5%) showed chronic inflammatory infiltration, while 17 (21.3%) exhibited mesothelial hyperplasia. Early atypical cellular architecture, suspicious for neoplastic transformation, was found in six cases (7.5%). Pathological analysis revealed that subjects from the hernia group presented with significantly higher rates of fibrotic thickening (p=0.021), vascular changes (p=0.034), and cellular atypia (p=0.011). All samples collected from the control group demonstrated no signs of malignant development.

Conclusion: The analysis of hernia sac tissue showed early signs that indicate potential pre-malignant changes under specific circumstances. Due to its capacity to spot early signs of cancer, the routine practice of microscopic evaluation enables better disease monitoring in surgical patients.

## Introduction

The surgical procedure of hernia repair stands as one of the most frequent operations that takes place globally in general and gastrointestinal surgery settings [1]. However, the evaluation of hernia sac tissue from these procedures occurs infrequently in diagnostic assessments, even though these operations are common [2]. Medical professionals traditionally view hernia sacs as harmless incidental findings before they discard the tissues without thorough examination after surgery [3,4]. Research evidence now indicates that hernia tissues contain early signs of pathology, such as chronic inflammation, together with possible pre-neoplastic and malignant changes [5].

Medical professionals should inspect hernia sac tissues because early indications of subclinical changes, including mesothelial hyperplasia, cellular atypia, and fibrotic structural modifications, may exist, albeit rarely [6,7]. The transformation patterns indicate initial neoplastic transformation in aging patients or those with long-standing hernia conditions [8]. Routine examination of hernia sac tissue - using surgical oncology methods alongside traditional histopathology - should improve early disease detection, thus allowing for better patient outcomes through early treatments and disease tracking [9].

This study aimed to explore the characteristics of hernia sac tissues obtained from elective procedures by analyzing them with tissues from control patients undergoing abdominal surgeries. The findings support the potential usefulness of routine histopathological analysis in hernia surgery, through which it reveals potential early-stage malignancies while advancing post-operative patient care.

## Materials and methods

This six-month prospective observational study included 100 participants during the period from January to September 2024. Eighty patients undergoing elective hernia repair operations were included in the study. The control group consisted of 20 patients undergoing different abdominal procedures whose demographic characteristics matched the study group patients by age and sex. This study was conducted in accordance with the Declaration of Helsinki, after informed consent was obtained from all enrolled patients. A priori power analysis was used to determine that 78 hernia patients and 20 control subjects would be required for the detection of a threefold difference in atypical cellular architecture rates, with the statistical power of 80% at a two-tailed α = 0.05. The sample size of this study met this requirement [10].

The study enrolled adult patients (≥18 years old) who had given written informed consent and had either an elective diagnosis of inguinal, umbilical, or incisional hernia or were going through elective cholecystectomy or appendectomy with intact peritoneum (control group). Patients were excluded if they had a history of any sort of malignancy, active systemic infection, or localized inflammation in the peritoneal cavity, or any other form of chronic inflammation, or if they were taking any form of immunosuppressant, or if they had any other intra-abdominal pathology. Surgeons extracted the hernia sac tissues right after surgery and preserved them in 10% formalin. Pathologists performed hematoxylin and eosin (H&E) staining for the histopathological assessment of the tissue samples. Two board-certified pathologists, blinded to group, evaluated each specimen based on inflammation, hyperplasia of mesothelial cells, fibrotic thickening, atypical cellular architecture, and blood vessel proliferation.

Multivariate logistic regression models adjusting for age, sex, BMI, and diabetes status were conducted for each histopathological feature to assess independent associations. Exact p-values were reported throughout. Continuous variables like age and BMI were presented as mean and standard deviation (SD). For comparison of these variables, independent t-tests were used. Categorical variables such as gender, status of diabetes, and all other features were compared using the chi-square (χ²) test. If any cell count was <5, then Fisher’s exact test was used. All tests were two-tailed. The Holm-Bonferroni method was used to control multiple primary comparisons involving five histopathological endpoints. An adjusted significance threshold of α = 0.01 was established for this cause. A p-value of <0.05 was considered significant for all other comparisons.

## Results

A total of 100 patients were enrolled, of whom hernia sac tissue pathology was evaluated in 80 patients undergoing hernia repair surgery and compared to 20 patients who had unrelated abdominal procedures (controls). Continuous variables such as age and BMI were evaluated by using the independent samples t-tests, and categorical variables such as gender and diabetes status (for immunological reasons) were analyzed using the chi-square test. No statistically significant findings were seen between the groups. Table 1 demonstrates these continuous variables.

**Table 1:** Demographic and clinical characteristics. P-value was calculated using the chi-square test. The t-test was used for continuous variables, and the chi-square test was used for categorical variables. Due to no difference between groups, the χ² value is 0 for both the 75% and 15% categories, respectively.

Variable	Hernia group (n=80)	Control group (n=20)	t-test (t) for continuous variables	Chi-square test (χ²) for categorical variables	p-Value
Age, mean ± SD (years)	55 ± 12	56 ± 11	-0.34	NA	0.75
BMI, mean ± SD (kg/m²)	26.8 ± 3.9	27.1 ± 4.2	-0.30	NA	0.77
Male sex, n (%)	60 (75.0%)	15 (75.0%)	NA	0.00	1.00
Diabetes mellitus, n (%)	12 (15.0%)	3 (15.0%)	NA	0.00	1.00

The characteristics of the hernia and control groups were comparable without any statistical significance. The hernia group was more prevalent in male patients, comprising 60 (75%) in the disease group and 15 (75%) in the control group, respectively. A pathological evaluation of the tissues examined, chronic inflammation, mesothelial hyperplasia, atypical cellular architecture, fibrosis, and vascular proliferation can be descriptively seen in Table 2.

**Table 2:** Histopathological findings in the study groups. Each variable was evaluated using the chi-square test or Fisher’s exact test when expected cell counts were below 5. Holm-Bonferroni correction was used for the five primary comparisons (adjusted α = 0.01). The results are given in the form of p-values and χ² values.

Pathological feature	Hernia group (n=80)	Control group (n=20)	Test statistic (χ²)	p-Value
Chronic inflammatory infiltration	34 (42.5%)	3 (15.0%)	5.64	0.018
Mesothelial hyperplasia	17 (21.3%)	1 (5.0%)	4.14	0.042
Fibrotic thickening	29 (36.3%)	2 (10.0%)	5.34	0.021
Vascular proliferation	22 (27.5%)	1 (5.0%)	4.50	0.034
Atypical cellular architecture	6 (7.5%)	0 (0.0%)	6.45	0.011

**Figure 1:**
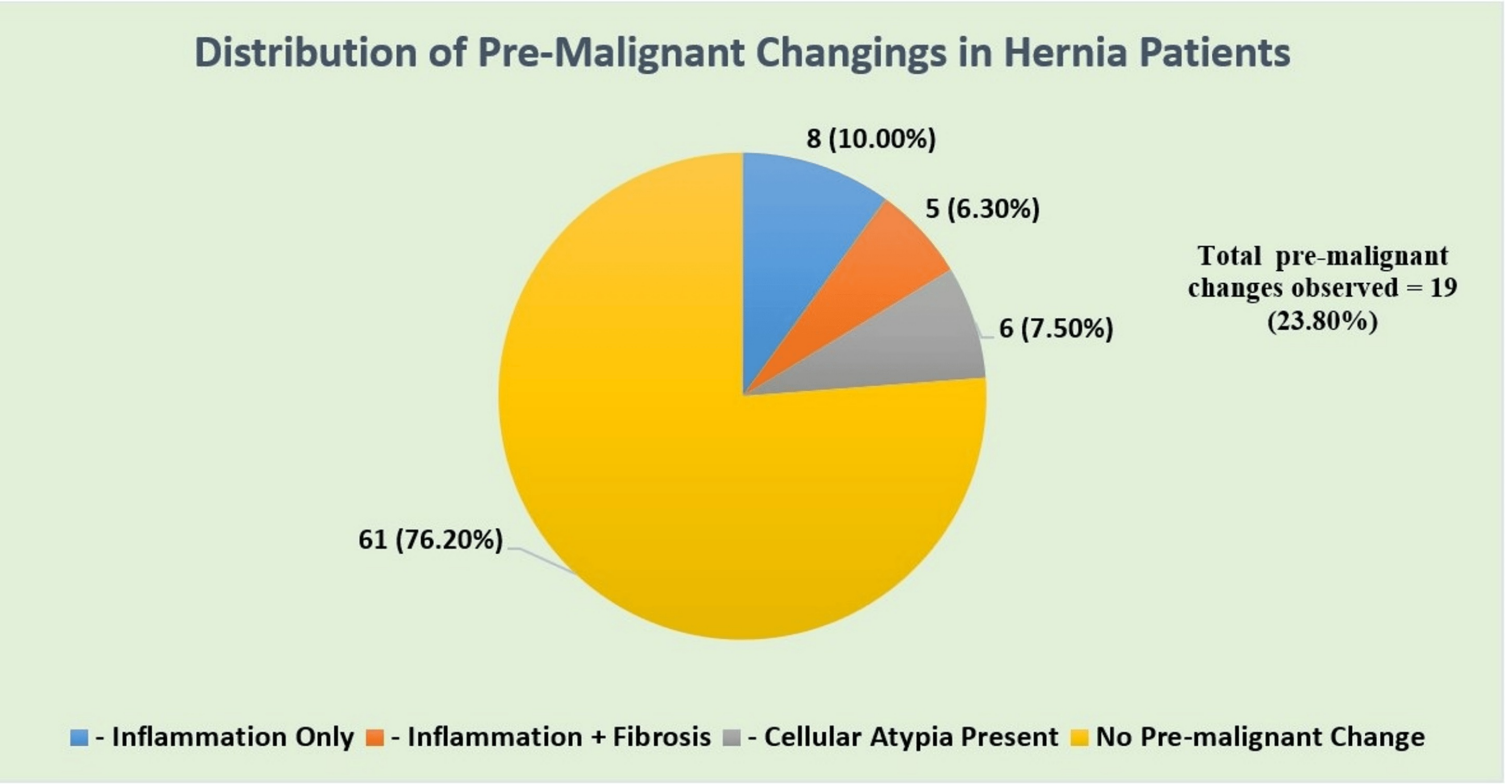
Distribution of pre-malignant indicators among hernia patients.

The patients with early neoplastic cellular abnormalities were six (7.5%). The most prevalent pathological findings were chronic inflammation and fibrotic changes. The mesothelial hyperplasia and vascular proliferation occurred more often in patients above 60 years of age; however, the outcomes were found statistically non-significant (p > 0.05). No statistical difference existed between the pre-neoplastic feature distribution among male and female participants. Figure 1 displays the frequency of pre-malignant histopathological features that were observed among the hernia group (n = 80). Features were classified on the basis of the presence of chronic inflammation, fibrotic thickening, and atypical cellular architecture.

Composite analysis of the early pre-malignant indicators inferred 19 (23.8%) of 80 (100%) hernia patients depicting any pre-malignant histopathological feature. Histological examination provided a sense for hernia evaluation because atypical cells appeared in six cases despite of benign clinical appearance. The lack of histopathological examination during standard hernia surgery procedures may lead to an unidentified diagnostic gap.

Multivariate logistic regression adjusted for age, sex, BMI, and diabetes highlighted some significant correlations, i.e., for fibrotic thickening (aOR: 4.75, 95% CI: 1.45-15.57, p=0.010) and vascular proliferation (aOR: 6.20, 95% CI: 1.52-25.32, p=0.011). Chronic inflammation (aOR: 3.92, 95% CI: 1.12-13.74, p=0.032) and atypical cellular changes (aOR: 8.15, 95% CI: 1.21-54.89, p=0.031) were also linked to hernia tissues. Mesothelial hyperplasia showed no significance (p=0.053). Fibrosis and vascular changes were observed to be independent predictors. These results gave valuable insights into histopathological changes in hernia for further research purposes.

## Discussion

Evidence from this study showed that surgical practitioners should pay attention to hernia sac tissues because they contain histopathological changes suggestive of early pre-malignant changes that are usually overlooked in routine tasks. Out of 80 patients undergoing elective hernia repair, pathological examination revealed chronic inflammatory infiltration in 34 (42.5%) patients, with mesothelial hyperplasia observed in over 17 (21.3%) cases. The examination identified cellular abnormalities within six (7.5%) tissue samples that may indicate early neoplastic potential.

Research results confirmed doubts about treating hernia sacs as nonessential clinical tissue, which has been a widely accepted practice for years. Multiple studies had documented the presence of mesothelioma, serous carcinoma, and metastatic deposits in hernia sacs, but these diagnoses were usually discovered following retrospective examinations [11]. Our prospective research conducted a detailed system of quantifying histopathological changes in this controlled cohort, which increased the clinical implications of these findings. Observations of fibrosis and increased vascular proliferation levels in hernia sac tissues surpassed those observed in control tissues, showing patterns that may resemble early-stage tumorigenic processes requiring stromal remodeling and progressive angiogenesis for cancer development [12]. New research confirmed that chronic peritoneal irritation leads to reactive mesothelial proliferation because chronic inflammation can potentially initiate mesothelioma development or serosal malignancies [13].

Relentless discomfort and chronic irritation in hernia sacs can stimulate abnormal mitotic activity through mesothelial proliferation, fibrotic remodeling, scar formation, and genomic destabilization. Chronic inflammation produces a set of formidable agents that include cytokines, mutagens, growth factors, and even reactive oxygen species (ROS), elevating the risk for mutations [14]. Although these molecular mechanisms were not directly studied in this study, cyclical physical damage followed by repair can lead to stromal angiogenic neovascularization remodeling, which are theoretically considered as precursor indicators of tumor formation. These events may integrate into oncogenic driver pathways such as NF-kB and JAK-STAT signaling cascades known for cell death and proliferation control. Such hypotheses may align with future molecular research [15].

Analysis of normal samples showed the absence of malignant or atypical elements, and this supports the theory that these findings exclusively occur in hernia tissue [16]. Research questions emerge about what factors, such as mechanical stress, hypoxia, and specific immune responses, possibly contribute to early malignancy development inside hernia areas. The current research changes the medical perspective by studying early and delicate histological alterations instead of focusing on late-stage cancer identification within hernia sacs, as previous studies investigated [17]. Standard hernia surgery procedures should include routine histopathological examination to maintain patient safety. Early detection of atypia or fibrosis helps doctors determine the risk level, mainly among elderly patients as well as those who experience hernia recurrence [18].

Despite the useful findings, this study had some limitations. The first one was a small control group and single-center design, which might prove inefficient for generalizability. Secondly, images of histopathological assessment based on the H&E staining were lost due to accidental damage in the diagnostic lab, which could enhance visibility of research, and third, long-term follow-up was required to assess the progression of the atypical changes. As an alternative, we have included a structured table summarizing the frequency and key features across groups, which in future aim to incorporate high-resolution micrographs for enhanced visual representations. Long-term research should establish whether these observed pathological changes serve as indicators of future cancer development, which would validate monitoring programs after hernia repairs.

## Conclusions

This study demonstrated that hernia sac tissue possesses diagnostic value, even though surgeons normally abandon it during surgical procedures. Routine microscopic evaluation of surgical specimens should be incorporated into the workflow after detecting initial histopathological indications, which include cellular atypia and mesothelial hyperplasia.

Clinical evidence implies that hernia tissues might serve as indicators for detecting early cancer, as well as for risk assessment. Researchers need to investigate molecular indicators, along with long-term follow-up outcomes, to develop clinical best practices for integrating histopathological examinations into standard hernia care.
